# The Role of the Coagulation System in Peripheral Arterial Disease: Interactions with the Arterial Wall and Its Vascular Microenvironment and Implications for Rational Therapies

**DOI:** 10.3390/ijms232314914

**Published:** 2022-11-29

**Authors:** Giuseppe Miceli, Maria Grazia Basso, Giuliana Rizzo, Chiara Pintus, Antonino Tuttolomondo

**Affiliations:** 1 Department of Health Promotion, Mother and Child Care, Internal Medicine and Medical Specialties (ProMISE), Università degli Studi di Palermo, Piazza delle Cliniche 2, 90127 Palermo, Italy; 2 Internal Medicine and Stroke Care Ward, University Hospital Policlinico “P. Giaccone”, 90100 Palermo, Italy

**Keywords:** peripheral artery disease, coagulation, atherosclerosis, anticoagulation

## Abstract

Peripheral artery disease (PAD) is a clinical manifestation of atherosclerotic disease with a large-scale impact on the economy and global health. Despite the role played by platelets in the process of atherogenesis being well recognized, evidence has been increasing on the contribution of the coagulation system to the atherosclerosis formation and PAD development, with important repercussions for the therapeutic approach. Histopathological analysis and some clinical studies conducted on atherosclerotic plaques testify to the existence of different types of plaques. Likely, the role of coagulation in each specific type of plaque can be an important determinant in the histopathological composition of atherosclerosis and in its future stability. In this review, we analyze the molecular contribution of inflammation and the coagulation system on PAD pathogenesis, focusing on molecular similarities and differences between atherogenesis in PAD and coronary artery disease (CAD) and discussing the possible implications for current therapeutic strategies and future perspectives accounting for molecular inflammatory and coagulation targets. Understanding the role of cross-talking between coagulation and inflammation in atherosclerosis genesis and progression could help in choosing the right patients for future dual pathway inhibition strategies, where an antiplatelet agent is combined with an anticoagulant, whose role, despite pathophysiological premises and trials’ results, is still under debate.

## 1. Introduction

Peripheral artery disease (PAD) is a clinical manifestation of atherosclerotic disease with a large-scale impact on the economy and global health, affecting over 200 million people worldwide [1]. The term PAD refers to all arterial trees, except for the aorta and coronary arteries, as recently emphasized by Tran et al. [2]. The prevalence of PAD is approximately 12% in the adult population, with males being affected slightly more than females [3]. Even if symptoms and manifestations may be disabling, most patients can present an asymptomatic PAD. Due to the lack of characteristic signs and symptoms, these patients are often under-recognized and undertreated [4].

The goal of identifying the molecular pathways involved in both asymptomatic and symptomatic patients is to prevent the progression and complications of PAD and find subjects with a high risk for cardiovascular disease [5]

In fact, though asymptomatic patients do not have limitations in carrying out daily activities, they present an increased risk for major cardiovascular events [6]. A large international registry of patients found that 5.4% of patients with established PAD had a major cardiovascular event such as cardiovascular death, myocardial infarction, or stroke at one year, and 21% experienced these endpoints or hospitalization for an atherosclerotic event [7,8].

Despite the role played by platelets in the process of atherogenesis being well recognized, evidence has been increasing on the contribution of the coagulation system to the atherosclerosis formation and PAD development [9,10].

In fact, the interaction between the activated platelet and the artery wall is a fundamental process for atherothrombotic disease, but it is also responsible for augmented fibrinogen concentrations, thrombin formation, and fibrin turnover. The role of coagulation proteins in PAD pathogenesis is established not only in acute thrombotic complications but also in the stable stages of the disease, where fibrinogen and thrombin seem to be associated with the clinical severity [11]. The growing evidence concerning the central role of the coagulation cascade in the pathogenesis of PAD is also responsible for important repercussions for the therapeutic approach.

Given the implication of the hemostasis process and coagulative proteins in the pathophysiology of PAD, the use of anticoagulant therapy alone or in combination with antiplatelets has been considered as a potential antithrombotic option for antiplatelets alone.

In this review, we analyze the contribution of the coagulation system and inflammation on PAD pathogenesis, focusing on molecular-pathway similarities and differences between atherogenesis in PAD—above all, the expression in lower extremity artery disease (LEAD) and coronary artery disease (CAD)—and discussing the possible implications for current therapeutic strategies and future perspective accounting for molecular coagulation targets.

## 2. Role of Coagulation in the Formation and Progression of Atherosclerosis

Coagulation is involved in thrombus formation and, therefore, in PAD progression. According to the composition, the thrombi can be categorized into acute or chronic (organized). Thrombus formation is driven by activated platelet and thrombin generation pathways. Acute thrombi are composed of red blood cells, fibrin, and platelets which adhere to the endothelial wall after the disruption of the atheromatic plaque and the exposure of the necrotic core. Chronic thrombus is defined by the presence of capillaries, smooth cells, connective tissue and inflammatory cells. While acute thrombi can be detected indiscriminately in advanced atherosclerotic and non-significant atherosclerotic lesions, chronic thrombi are observed more frequently in non-significant atherosclerotic plaque.

The disruption of an atherosclerotic lesion, exposing thrombogenic material to the blood, is the starting mechanism of the atherothrombotic events leading to plaque progression [12]. Several coagulation proteins have been implicated in proinflammatory conditions and atherosclerosis. The presence of tissue factor (TF) is considered the primary physiologic trigger of the coagulation cascade in atherosclerotic lesions: Wilcox et al. showed that TF was found on the membrane of macrophages and vascular smooth muscle cells, where it co-localizes with factor VII [13]. Furthermore, elevated TF levels have been found in patients with PAD. Abnormal TF levels have been detected mainly in early atherosclerotic lesions compared to stable advanced atherosclerotic plaque, suggesting a procoagulant state, especially in early stages of atherosclerosis, amplified by the reduction of inhibitor pathways of coagulation, as demonstrated by a higher TF/TF pathway inhibitor ratio in patients with early atherosclerosis [14].

Endogenous thrombin and thrombin–antithrombin complex values indicated a procoagulant profile of early atherosclerotic lesions compared to stable advanced atherosclerotic lesions. In subclinical atherosclerotic disease, a relation between TF and an increased carotid intimate media thickness has been documented as a marker of early atherosclerosis [14]. The presence of coagulation components, such as thrombin levels and thrombin/antithrombin complexes, in atherosclerotic lesions suggests the role of these clotting proteins in plaque thrombogenicity. The fact that coagulation proteins are more present in early atherosclerotic lesions compared to advanced atherosclerotic lesions supports an important role for these coagulation factors in the initial development of atherosclerosis rather than an involvement limited to thrombus formation in unstable plaques only [15].

These data might suggest a possible thromboembolic phenomenon in the peripheral arterial occlusion—above all, in infrapopliteal arteries—explaining the relatively lower incidence of chronic limb ischemia compared to PAD prevalence [16].

These data support the evidence of a procoagulant state in patients with peripheral artery disease, whose expression is more evident in the early stages, suggesting that thrombotic complications do not depend on the plaque size.

Furthermore, abnormal levels of thrombin, factor X, TF, and FXII have been detected in circulating blood. The expression of these proteins has also been noticed on the arterial wall, particularly in the early stages. This different expression might be attributed to the different plaque compositions. Indeed, early plaques are composed of inflammatory cells, producing procoagulant proteins, so the expression of coagulation factors in the vascular wall depends not only on the translocation from blood flow but also on the local production by the inflammatory cells. A change in plaque composition might explain why stable advanced atherosclerotic lesions, composed of fibrotic tissue surrounding the necrotic lipidic core, express fewer coagulation factors [17,18].

## 3. Plaque Composition and Vulnerability: Differences in Coronary and Peripheral Atherosclerotic Disease

The modified AHA classification [19] categorizes atherosclerotic plaques into adaptive intimal thickening (AIT), pathological intimal thickening (PIT), fibroatheroma (FA), and fibrocalcific plaque (FC). AIT is considered the expression of non-significant atherosclerosis, and it also includes atherosclerotic lesions composed of smooth cells without a lipid matrix. PIT refers to plaques with smooth cells and extracellular lipids, while fibrous caps surrounding lesions are classified as FA and calcified plaques are classified as FC; PIT, FA, and FC are classified as significant atherosclerosis.

CAD and PAD might be considered two distinct expressions of the same condition, but, although similar, they present different characteristics that are secondary to the peculiarities of the two vascular districts affected. Many factors, including anatomical features such as vessel size and tortuosity, local shear stress and inflammation on the arterial wall, and the presence of vasa vasorum, are responsible for a different composition of atherosclerotic plaques in coronary and peripheral arteries. In fact, coronary lesions are prevalently constituted from a central lipid-necrotic core composed of cholesterol (LRNC), oxidized LDL, and necrotic foam cells surrounded by a thin fibrotic cap, while the LEAD plaques present more frequently fibroproliferative and calcific lesions, without q lipid core and a prevalent composition of collagen, smooth cells, and calcification [20].

The different plaque composition is responsible for more vulnerability in the coronary than the peripheral arteries. Indeed, plaque stability depends on the percentage of fibrotic and lipidic components; therefore, connective tissue stabilizes atherosclerotic lesions, making complications, such as plaque rupture, less likely [21].

Many studies have analyzed the plaque composition in coronary and peripheral arteries with non-invasive imaging such as Magnetic Resonance, proving that only 25% of patients with LEAD had LRNC lesions, with a higher prevalence in smoking patients [22,23]. LRNC plaques are associated with a higher risk of LEAD complications, defined as a worsening ankle brachial index (ABI) and critical limb ischemia requiring intervention, [22] even if prior studies did not find any significant association between LNRC and ABI decline or lumen stenosis but rather only with local atherosclerotic severity [24].

The risk of complications also depends on the increase in inflammatory cells in the plaques. Recent evidence has demonstrated a major prevalence of macrophages and lymphocytes in the coronary and carotid lesions compared to the femoral plaques, supporting a less inflammatory process in peripheral circulation, as confirmed by a lower uptake of fluorodeoxyglucose in the femoral arteries compared to the carotid arteries in many studies using FDG-PET to determine inflammatory involvement in the atherosclerotic disease [25,26].

Histopathological studies have shown that atherosclerotic disease progression is related to local clotting formation. Some plaque components—in particular, cholesterol compounds—reveal marked thrombogenicity, promoting the activation of the coagulation cascade and the thrombus formation.

Previous studies demonstrated abnormal concentrations of coagulation factors, such as von Willebrand’s factor, fibrinogen, and factor VIIa, as well as increased plasminogen activator inhibitor-1 and tissue-type plasminogen activator antigen in patients with CAD, promoting the hypothesis that an impaired balance between coagulation and fibrinolysis might be involved in coronary atherosclerosis. [27]. These data were confirmed in more recent studies, underlying the possible role of some clotting proteins as biomarkers of CAD. In fact, elevated levels of fibrinogen and soluble fibrin monomer complexes and decreased concentrations of prothrombin were identified in patients with CAD in comparison with healthy controls [28]. As opposed to cardiovascular events, which occur in the case of plaque rupture, peripheral ischemia is often associated with chronic thrombus formation in the absence of atherosclerotic lesions, indicating a possible procoagulant state in PAD. Coagulation plays an essential role in the initial processes leading to plaque formation but also in the growth of the atherosclerotic lesions and the progression of peripheral artery disease, as suggested by the finding of altered levels of coagulation factors in patients affected by PAD [16].

## 4. Endothelial Disfunction in Chronic Kidney Disease and the Risk of Lower Extremity Atherosclerosis

Patients with kidney disease are more often diagnosed with peripheral atherosclerosis than equivalents without renal dysfunction [29,30,31]. Chronic kidney disease (CKD) and atherosclerosis share the same risk factors (smoking, age, sex, comorbidities such as hypertension, diabetes, and hyperlipidemia) inducing vascular damage. Nevertheless, different studies have demonstrated that kidney dysfunction is associated with a higher risk of peripheral artery disease regardless of the classical risk factors for atherosclerosis [32,33,34,35,36].

There are two main mechanisms responsible for endothelial dysfunction in patients with chronic kidney disease. The first one is the activation of a proinflammatory state: indeed, patients affected by end-stage renal disease (ESRD) show elevated serum concentrations of acute phase proteins, such as C-reactive protein, IL-6, and TNF-alpha, demonstrating the activation of proinflammatory processes in kidney injury [37]. On the other hand, the reduced availability of molecules with vasodilatory action—in particular, nitric oxide (NO) bioavailability—induces the dysregulation of the vascular tone. Confirming these data, recent studies showed impairment in endothelium-dependent vasodilatation in ESRD [38].

Inflammation causes endothelial dysfunction, inducing the expression of adhesion molecules on the vascular wall and increasing the levels of TNF-alpha, which are responsible for the cytotoxic effect on the endothelial cells [39]. As the renal damage progresses, some soluble adhesion molecules, such as ICAM-1, VCAM-1, and MMP, are dismissed from endothelial cells and activate the NfkB pathway, with the final result of reducing the NO levels [40].

Oxidative stress is another mechanism that is decisive for endothelial dysfunction in chronic kidney disease: the interconnection between these two processes comes through increased levels of myeloperoxidase (MPO). Indeed, the activation of neutrophils in inflammatory processes determines the production of MPO, using NO as a substrate, and leads to lipid peroxidation through the generation of diffusible oxidants and oxidized-LDL (ox-LDL), whose levels in CKD are inversely related to endothelial function [41].

Moreover, oxidative stress leads to the production of the AGE (advanced glycation end-products), molecules whose action is to inhibit DDAH, and the degradation enzyme of asymmetric dimethylarginine (ADMA), a competitive inhibitor of L-arginine inducing the inactivation of eNOS (endothelial NO synthase), with a consequently reduced availability of NO [42].

Increased levels of AGE are detected in patients with chronic kidney disease.

In addition, the lower serum concentration of triiodothyronine induced by inflammation is another factor responsible for impaired ADMA levels in ESRD [43].

Other factors cause the lowering of NO availability in CKD patients. Hyperphosphatemia, vitamin D deficit, and FGF23 overexpression contribute to the inactivation of eNOS in renal damage; furthermore, the lower levels of NO in renal dysfunction are also the consequence of the reduced synthesis and transport of the precursor L-arginine [44,45,46,47].

A recent study by Batkoa et al. showed an elevated expression of thrombomodulin (TM) in patients with ESRD [48]. TM is a glycoprotein whose expression has been found on the endothelium covering atherosclerotic plaques and on the macrophages and smooth cells; TM is also released from endothelial cells after vascular damage [49,50]. The increased levels of TM are positively associated with TNFR2 (soluble tumor necrosis factor receptor type 2) and osteopontin (OPN) expression. TNFR2 activation induced by TNFalpha binding has a central role in the clotting pathway beginning, leading to arteriolar thrombosis [51], while osteopontin is a cytokine involved in differentiating smooth muscle cells into an osteogenic phenotype, promoting vascular calcification and the progression of atherosclerotic disease [52,53]. OPN concentrations correlate with medial arterial calcifications, which are associated with a higher risk of cardiovascular and mortality risk in patients with CKD [54]. However, the mechanisms through which OPN and TNFR2 are related to increased levels of TM still need to be clarified.

Finally, increased concentrations of vWF (von Willebrand Factor) have been detected in patients with ESRD, suggesting a correlation between the coagulation system and thrombogenicity in kidney disease [55,56].

The interconnection between chronic kidney disease and endothelial dysfunction led to defining a new life-threatening clinical entity called malnutrition-inflammation-atherosclerosis (MIA) syndrome, in which the three aforementioned mechanisms synergistically concur to increase the cardiovascular risk and set the outcome of ESRD patients [57].

Based on these data, we can affirm that endothelial dysfunction in chronic kidney disease results from different processes leading to endothelial damage and dysregulation, which are responsible for accelerated atherosclerosis and a higher incidence of lower extremity atherosclerotic disease in patients with renal impairment.

About a quarter of patients with CKD with eGFR < 60 mL/min/1.73 m^2^ suffer from peripheral artery disease, representing an independent risk factor for mortality and extended hospitalization, particularly in chronic limb ischemia [58].

The incidence of LEAD (Lower Extremity Atherosclerotic Disease) is higher in proportion with the degree of kidney damage: the risk of LEAD is 1.2–2.5 fold higher in patients with chronic kidney disease G3–G5, and the risk of lower limb complications needing amputation is more frequent in eGFR < 30 mL/min/1.73 m^2^ in comparison with patients with normal renal function [59,60]. In addition, the risk is higher in patients undergoing dialysis because of the biochemical abnormalities leading to systemic inflammation, hypoalbuminemia, and hyperphosphatemia associated with chronic uremia [61,62].

The demonstration that CKD is a significant risk for LEAD is supported by data coming from a recent meta-analysis evaluating the outcome in patients with renal damage undergoing peripheral artery interventions in comparison with patients not affected by kidney disease: the study showed that target lesion revascularization (TLR), major amputations, and long-term mortality were most frequent in patients with CKD and, in particular, in ESRD, suggesting a pathogenic role of kidney dysfunction in the severity of peripheral atherosclerosis [63].

The data reported can lay the basis for the optimal clinical approach to patients with CKD who need to be evaluated for asymptomatic peripheral artery disease through the measurement of the ankle-brachial-index (ABI) to prevent the progression of lower extremity atherosclerosis and complications, even if ABI might be falsely normal in patients with ESRD and medial arterial calcification such that other diagnostic tools such as exercise ABI, the toe-brachial index, or duplex ultrasonography should be performed.

Lower extremity atherosclerosis in CKD is, therefore, the result of a complex interlacement of multiple processes resulting in vascular damage: for this reason, the management should include the participation of a multidisciplinary team, including a vascular surgeon, a physician, a wound care specialist, and a nephrologist, to perform the best diagnostic and therapeutic assessment of patients with peripheral atherosclerosis and chronic kidney disease.

## 5. Pad: An Inflammatory or a Procoagulant State?

Atherosclerosis has always been considered an inflammatory disease. However, as inflammation is an essential pathogenic mechanism in developing plaques, recent studies have demonstrated that other pathways occur in atherosclerotic disease. The expression of coagulation factors on the surface of the inflammatory cells suggested a fundamental role of the coagulation cascade in the onset of thrombotic complications and plaque formation.

Several studies reported the abnormal expression of thrombin, factor X, TF, and FXII in atherosclerotic plaques. Interestingly, the expression of coagulation factors has been found not only in advanced atherosclerotic disease but mainly in early atherosclerosis, such as in intimal media thickening, suggesting a pathogenic role of coagulation in atherogenesis [14,64].

**Figure 1:**
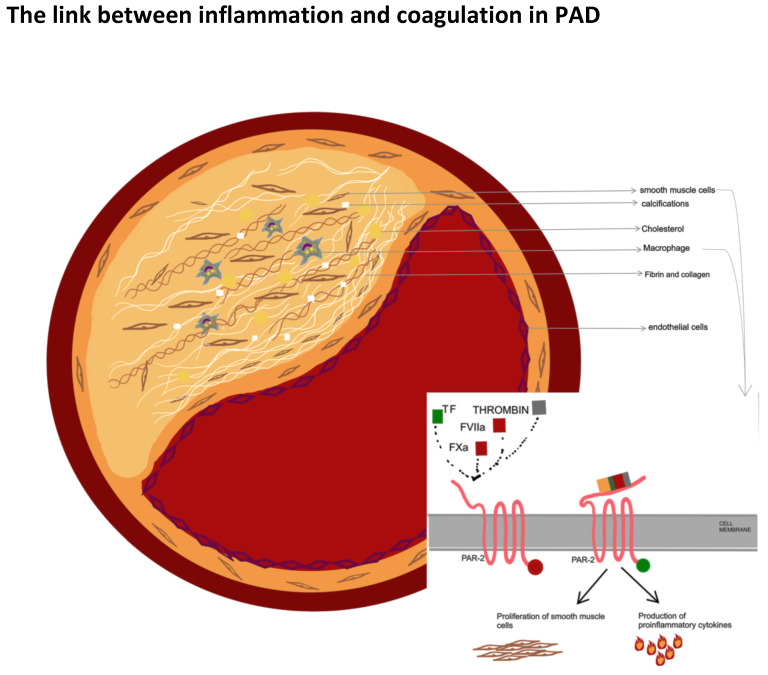
The picture shows PAD’s plaque composition that is classically not characterized by a lipidic core but is instead reached in fibrin, collagen, and smooth muscle cells. Macrophages and smooth vascular cells express PARs (protease-activated receptors) and Gprotein-coupled receptors, whose activation by factors implicated in the coagulation cascade (i.e., TF, FXa, FVIIa, and thrombin) leads to the production of proinflammatory cytokines, thus establishing a link between coagulation and inflammatory milieu. PAD: Peripheral Artery Disease; TF, Tissue Factor; FXa, activated Factor X; FVIIa, activated Factor VII; PAR-2, protease-activated receptor-2.

Coagulation factors—in particular, TF, FVIIa, factor Xa, and thrombin—have been considered inductors of inflammatory pathways through protease activated-receptors (PAR)-signal activation (Figure 1).

The PARs are G-protein-coupled-receptors whose expression has been found on human platelets, macrophages, and smooth vascular cells. Four PARs have been identified (PAR1, PAR2, PAR3, PAR4), and PAR1 and PAR2 are the most investigated in atherogenesis. Even though the pathogenic mechanism through which the PAR-signal induces atherosclerotic disease is not yet fully understood, different hypotheses have been proposed.

PAR-2 activation, in association with other co-factors such as IL-1b and tumor necrosis factor, induces the proliferation of smooth muscle cells and, through an MT-SP1/matriptase complex, the production of proinflammatory cytokines (such as IL-6 and IL-8). Furthermore, PAR2 leads to leukocyte adhesion to the vascular wall; in coronary arteries, PAR2 induces the activation of pathogenic pathways involved in inflammation, such as NfKb signaling, and the production of cyclo-oxygenase. Finally, PAR2 modulates the vascular tone and, consequently, blood pressure [65,66,67,68].

PAR-1 activation leads to the production and release of several factors (ADP, Thromboxane, p-selectine) and provokes platelet activation and aggregation; PAR-1 is also responsible for endothelial damage, which is one of the initial steps of atherogenesis. The role of PAR-4 in atherosclerosis is still unknown, as it seems to play a co-factor in the thrombin-induced PAR-1 pathway [68].

Several studies using animal models attested to the link between PAR pathways and atherosclerosis, as well as a genetic deficit of PAR2 that correlates with less of a risk of the progression of atherosclerosis [69].

According to Riewald et al., thrombin catalyzes PAR1 and PAR4 activation and clivation, while the TF-VIIa complex mediates PAR2 activation [70].

## 6. COVID-19: A Potential Model of the Hyperinflammation and Dysfunction of Coagulation

Coronavirus Disease-19 (COVID-19) is characterized by a hyperinflammatory state which begins with viral replication and culminates in an inflammatory-driven phase defined by the production and release of inflammatory cytokines and chemokines by infected cells (alveolar pneumocytes, epithelial cells, and macrophages). These events perpetuate an inflammatory response through the attraction of other circulating inflammatory cytokine-producing cells, such as neutrophils, natural killer cells, and T-lymphocytes [71,72,73,74,75].

The role of inflammation as a pathogenetic factor of COVID-19-associated morbidity and mortality has hence been studied both from a molecular and from a clinical-practice point of view, using anti-inflammatory therapies, such as glucocorticoids and IL-6 inhibitors, and reporting their improving effects on mortality and organ complications in severe COVID-19 patients [76,77,78,79].

Janus Kinases (JAK) Signaling has a central role in triggering inflammation, since many cytokines signal through a receptor activation-induced JAK signalling cascade [80]. In turn, activated JAKs control the activation of signal transducers and activators of transcription (STAT), which are responsible for the expression of genes and regulatory proteins implicated in various roles, such as cellular proliferation, differentiation, and immune response. For instance, IL-6 is one of the cytokines produced during the COVID-19 hyperinflammatory state, and its binding to the IL-6 receptor activates *JAK–STAT* signaling in various cells, including endothelial and inflammatory cells, thus taking to the release of chemokines and promoting monocyte and neutrophil recruitment [81]. Moreover, activated *JAK–STAT* upregulates several thrombotic factors such as tissue factor, vWF, and angiopoietin-2, and is hence linked to the upregulation of the extrinsic coagulation cascade and therefore contributes to macro- and microvascular thrombosis [73]. These processes lead to a systemic inflammation which makes COVID-19 a systemic inflammatory disease surely characterized by a peak of severe acute inflammation held by an altered immune response and potentially chronic inflammation even after the resolution of the acute phase of the disease [82].

This proinflammatory-procoagulant self-sustaining vicious circle is the foundation of COVID-19 patients’ increased cardiovascular morbidity and mortality, as reported by several studies. In particular, a few small studies have reported increased elastic artery stiffness and arterial dysfunction in the early post-acute phase of COVID-19 [83,84,85,86,87]. Increased arterial stiffness could be linked to various possible mechanisms such as changes in arterial wall biomaterials, which is itself also determined by the abovementioned hyperinflammatory state: for example, studies have shown that interleukin 1, a cytokine involved in inflammation and in the arterial stiffening in patients with inflammatory diseases, could play a role in determining these effects in COVID-19 patients, since it leads to changes in the phenotype of vascular smooth muscle cells [88].

Zanoli et al. [89] found that, in COVID-19 patients, carotid distensibility was slightly improved during follow-up, suggesting that elastic arteries’ stiffness following the acute phase of infection could be reduced, although not fully reverted. Nevertheless, it is still under debate whether the residual structural damage of the arterial wall is due to a chronic low-grade inflammatory milieu persisting after the acute phase of the infection. Larger and longer follow-up studies are needed.

## 7. The Role of Inflammation and the Immune System in Atherosclerosis

Atherosclerosis has classically been defined as an immune-inflammatory disease based on the immune system and inflammatory pathways converging to determine endothelial damage, which constitutes the initial stage of atherogenesis. The intersection of the two abovementioned systems carries the final result of perpetuating the production of pro-inflammatory molecules and the recruitment of immune cells, leading to the formation and growth of the plaques in the vascular wall.

### Involvement of the Immune System in Atherosclerosis

**Innate immune system**. The innate immune system consists of monocytes–macrophages, neutrophils, and dendritic cells, whose role in different moments of atherogenesis is well-known.

Increased levels of monocyte in the plaques have been detected in mice models with a high-fat content diet. Evidence suggested that the amount of monocyte cells in the atherosclerotic lesions correlates with peripheral blood monocytosis, suggesting that it may be a potential risk factor for atherogenesis [90].

The monocytes migrate into the intima and adventitia layers from peripheral blood due to the action of specific chemokines and turn into dendritic cells and macrophages.

Among the different types of resident endothelial wall macrophages, those with the ability to secrete elevated amounts of IL-1β are associated with a higher risk of development and the growth of atherosclerotic plaques, as many studies conducted on animal and human models have confirmed [91].

Concerning dendritic cells, three subtypes with different proatherogenic functions have been described. In peripheral blood, plasmacytoid dendritic cells induce inflammation through the production of proatherogenic cytokines (TNF, IL6, IL12, INF). In contrast, type 1 and type 2 dendritic cells located in lymphoid and non-lymphoid sites are engaged in the recruitment of cytotoxic cells and adaptative immune T-cells after the production of CCL19 and CCL21 chemokynes, respectively [92,93].

Dendritic cells and macrophages are able to uptake lipidic products, turning into foam cells, the main compounds of fatty streaks and atheroma [94,95,96,97].

Neutrophils are involved in endothelial damage because of the capability of secerning reactive oxygen products and neutrophil extracellular traps, molecules that are able to recruit and activate macrophages into the arterial wall [98,99].

**Adaptative immune system.** T-cell and B-cell lines are both implicated in atherogenesis.

Several studies showed the T-cell involvement in the beginning and progression of atherosclerosis, underlying the different roles of each cell subtype in the risk for the development of atherosclerotic plaques. While the pro-inflammatory action of T helper 1 (TH1) cells has been well-confirmed, the proatherogenic function of TH2 cells and TH17 cells is still unclear [100].

Not all of the CD4+ T-cell subtypes present inflammatory activity. CD4+Treg cells have an anti-inflammatory function, which derives from the inhibitory action on the Th1-cell activity, the attraction of circulating monocytes into the endothelial wall, and the proliferation of dendritic cells [101,102].

A controversial role has been awarded to CD8+ T-cells: atherosclerotic lesions show the accumulation of CD8+ T-cells secerning INFγ, suggesting their contribution to the atherosclerotic processes. Conversely, the activation of a B-cell pathway provides a protective role of CD8+ T cells [101].

The role of B-cells in the atherosclerotic process is less noticeable than that of other immune cells: B2 cells carry out their supposed atherogenic role by inhibiting T-follicular helper cells and activating an immunoglobulin-mediated inflammatory response in the lymphoid organs [103].

## 8. Inflammatory Cytokines in the Atherosclerotic Disease

The roles of several cytokines in atherogenesis have been extensively documented.

IL-1, with both isoforms, is one of the central cytokines implicated in the deployment of atherosclerotic plaque formation. IL-1α has been implicated in the remodeling of the arterial wall. IL-1β results from the precursor pro-IL1β undergoing clivation mediated by the caspase-1 after NLRP-inflammasome activation, and it is considered to be the principal activator of many different inflammatory pathways, carrying the formation of atheroma.

IL-1β promotes the secretion of other cytokines, such as IL-6, which promotes acute phase protein production (CRP, fibrinogen) [104].

The essential role of IL-1 in atherogenesis has been assumed by several studies, proving that IL-1 levels are related to the severity of atherosclerotic disease. This hypothesis has been supported by studies performed on IL1-knockout mice, which have shown a reduction in the incidence and size of atherosclerotic plaques compared with the control group not exhibiting the deficit of the cytokine [105].

Moreover, the IL-1β blockade led to a less inflammatory phenotype of monocyte, decreasing the plaque size [106].

IL-18 is another cytokine which acts as a trigger in inflammatory pathways. Like IL-1, active IL-18 derives from a pro-active form, which undergoes cleavage mediated by caspases. The expression of IL-18 can be found on the surface of different cell types involved in the atheroma development, such as endothelial cells, macrophages, and smooth muscle cells.

As for IL-1, several studies based on animal models have demonstrated the pathogenic effect of IL-18 on the vascular damage, leading to atherosclerotic plaques formation. Interestingly, in a study performed by Mallar et al., mice overexpressing IL-18BP showed more severe atherosclerotic disease compared with models with deficiency of the cytokine [107]. The proatherogenic role of TNF-α is carried out by the expression of adhesion molecule VCAM-1 on the endothelial cells, favoring the migration of inflammatory cells in the vascular wall [108,109].

Data supporting this proatherogenic action come from studies on animal models where the lack of TNF seemed to protect from atherosclerotic disease. Moreover, some other studies supported the hypothesis of a potential role of TNF-α in the prediction of cardiovascular events [110,111,112,113].

TNF secretion is induced by INFγ, which explicates its proatherogenic role through other mechanisms, including the release of metalloproteinases, the production of ROS (reactive oxygen species), and the leukocyte migration from peripheral blood [91,114].

## 9. Coagulation Factors as Markers of Risk in Peripheral Artery Disease

Given the interconnection between the pathogenesis of PAD and coagulation, several studies have been conducted in order to evaluate abnormal values of clotting factors in patients affected with peripheral artery disease compared to the general population: in particular, some studies found a correlation between PAD and fibrinogen, D-dimer antigens, von Willebrand factor, and tissue plasminogen activator (tPA), whose levels appear to be higher in patients with PAD than in healthy subjects [115,116,117].

### 9.1. Tissue Factor and Tissue Factor Pathway Inhibitor

TF, also known as tissue thromboplastin and expressed on the surface of the platelets, monocyte cells, and the subendothelial tissue, is a 44 kDa glycoprotein that is crucial in the activation of the coagulation pathway. The extrinsic way of coagulation begins with the exposition of TF after vascular damage. TF binds and activates factor VII in VIIa, triggering the coagulation cascade and the clot formation. Furthermore, TF may be detected in a soluble form in the plasma, and its release might be induced by several factors, such as inflammatory cytokines or procoagulant states. Some studies tried to relate tissue factor levels with the risk of vasculopathy.

Several years ago, Blann et al., evaluated the concentration of tissue factor in the plasma of patients with PAD and healthy people, finding that TF levels were higher in the first group compared to the latter; in particular, this difference was statistically significant considering young people among the PAD group, pointing out a likely independent role of this coagulation factor in the development of peripheral artery disease, regardless of other possible confounders in the elderly patients [118].

Recently, Radosław et al. studied the difference in the concentration of TF in patients with chronic limb ischemia and intermittent claudicatio in comparison with healthy people; they confirmed higher concentrations of TF in patients with LEAD and, in particular, in the subgroups of patients with chronic ischemia whose TF concentration was significantly increased compared to patients with intermittent claudicatio [119].

The activity of TF is modulated by the tissue factor pathway inhibitor (TFPI): it is a 42 kDA glycoprotein inactivating factor Xa and then the TF/factor VIIa complex, interrupting the coagulation cascade. A potential role of this protein in atherosclerotic disease has been supported by the discovery of the expression of TFPI and the reduced TF activity in carotid plaque [120], while increased concentrations of TFPI were detected in coronary artery disease [121]. TFPI may be present in the plasma in two forms: free TFPI and TFPI bound into the TFPI/FXa complex. No differences in the free TFPI concentrations have been found between PAD and healthy people, though patients with PAD have higher total TFPI levels than the control group [122]. TFPI and TF activities are inversely correlated, even if it is not clear if the elevated TF levels influence the decreased concentration of TFPI or vice versa.

### 9.2. Fibrinogen

Fibrinogen is another crucial protein for clotting formation. Even if most studies were conducted to understand the role of this protein in the coronaropathies, suggesting that elevated levels of serum fibrinogen can be related with a high risk of acute cardiovascular events [123], recent studies turned to the potential role of this protein as a marker of risk in PAD.

Some studies demonstrated increased fibrinogen levels in the serum of patients with PAD [124]. Considering the high cardiovascular morbidity and mortality in patients with PAD, some studies tried to verify the possible role of fibrinogen levels as an outcome predictor in patients with PAD [125].

Altes et al. conducted a study using the data from the FRENA (Factores de Riesgo y ENfermedad Arteria) registry in order to find a possible correlation between plasma fibrinogen and mortality in patients with symptomatic PAD in comparison with healthy people. They evaluated the different incidence of the primary outcome (defined as myocardial infarction, ischemic stroke, or limb amputation, major bleeding, or death during the study period) into the two groups and the correlation with raised fibrinogen levels, defined as values > 450 mg/100 mL. At the end of the observational period, 41% of patients with PAD recruited showed elevated fibrinogen levels. These patients had an increased incidence of the primary endpoint, suggesting that elevated levels of fibrinogen in patients with PAD are predictive of the risk for the development of ischemic events (HR: 1.61; 95% CI: 1.11–2.32) or major bleeding (HR: 3.42; 95% CI: 1.22–9.61), while no differences have been noticed on the risk of death. Thus, fibrinogen might be considered an additional risk factor for the development and progression of PAD, potentially opening new therapeutic strategies for the prevention of PAD complications [126].

### 9.3. D-dimer

The excessive formation of thrombi in the blood circulation is counteracted by the activation of fibrinolysis, the opposite process of coagulation which leads to clot dissolution. This process starts when tPA is activated after binding the plasminogen, which turns into the activated form of plasmin. Plasmin is an enzyme involved in the dissolution of the thrombus, with a consequent release of degradation products such as fibrin. One of the markers of fibrinolysis is D-dimer.

In one study by Sara Arfan et al., elevated concentrations of Prothrombin Fragment and D-dimer were detected in patients with LEAD compared to the control group. Furthermore, this composite marker has been related to a higher risk for disease progression and the development of complications. Patients with LEAD and higher levels of prothrombin fragment and D-dimer showed ABI decline and cardiovascular events incidence in the follow-up at two years, suggesting a possible role of the combination of Prothrombin Fragment and D-dimer as a predictor of poor prognosis in LEAD [127].

One more confirmation about the helpful role of D-dimer as a marker for the stratification of risk in patients with PAD is contained in the BRAVO study, which correlated the levels of D-dimer in the serum of patients with PAD and the occurrence of cardiovascular events by collecting blood samples every two months. This trial found elevated levels of D-dimer in the blood samples of patients with PAD within two months before an ischemic heart disease event, and the difference was statistically significant compared to the level of the marker in the samples obtained previously [128].

### 9.4. Von Villebrand Factor and ADAMTS13

Von Willebrand’s factor (VWF) is a large glycoprotein synthesized and secreted by endothelial cells to form the subendothelial matrix. After vascular damage, it is exposed to blood circulation, promoting platelet adhesion to the subendothelial matrix through the receptor Glycoprotein Ib/IX; soluble VWF binds FVII, preventing the degradation of this molecule.

VWF is a 220 kDalton monomer, but a process of multimerization is responsible for the activation. This process is regulated by a disintegrin-like metalloprotease with thrombospondin-type 1 repeats, member-13 (ADAMTS-13), which cleaves multimeric VWF into inactive fragments.

VWF and ADAMTS13 have an essential role in platelet aggregation and the coagulation pathway: an impaired balance between these two molecules has been found in some atherosclerotic and inflammatory diseases [129]. Elevation in the plasma levels of VWF is associated with arterial thrombosis, so it has been proposed that lower concentrations of ADAMTS13 might be associated with a higher risk of cardiovascular events. The data in this regard are conflicting, since some studies have shown a relationship between an elevated risk of myocardial ischemia and low levels of ADMTS13 [130,131]. At the same time, one trial demonstrated a proportional correlation between the risk of coronary artery diseases and ADAMTS13 plasma concentrations [132]. These controversial data may result from bias, such as elderly age or medications, which could invalidate ADAMTS13 levels and the risk of events.

Lower levels of ADAMTS13 have been detected in patients with thrombocytopenic thrombotic purpura, favoring the microangiopathic damage.

The VWF/ADAMTS13 ratio has been proposed as a cardiovascular risk marker for patients with PAD. In one study by Green et al., VWF and ADAMTS13 levels were measured in two groups of patients presenting cardiovascular events; while there were no differences in the concentrations of the molecules between PAD patients and controls, the VWF/ADAMTS13 ratio was higher in patients with peripheral artery disease two months before the acute event, even if the difference was not statistically significant [133].

Polok et al. demonstrated that vWF levels are correlated with the risk of LEAD progression and the development of complications after revascularization: increased concentrations of vWF were detected in patients with thrombotic complications three months after open or endovascular revascularization [134].

Furthermore, a recent randomized controlled trial has shown elevated levels of vWF in induced DM/HC (diabetes mellitus/hypercholesterolemia) swine, which developed atherosclerosis, suggesting that vWF might be a potential marker of endothelial dysfunction in people with metabolic syndrome [135,136].

### 9.5. Tissue Plasminogen Activator

tPA is a 70 kDa protein involved in converting plasminogen into the active plasmin, which is responsible for the dissolution of clots.

Previous studies have shown a correlation between tPA levels and coronary artery diseases, [137,138,139,140,141] while limited data are available on this molecule’s role in other vascular regions.

A recent study was performed by evaluating the tPA concentrations in a group of 80 patients with symptomatic lower extremity artery disease compared to 30 healthy people, finding elevated tPA-Ag levels in the first group, with a statistically significant difference. Elevated levels of this molecule indicate endothelial damage, since endothelial cells are the main productors. Moreover, elevated tPA levels contribute to the form of inactivated tPA-PAI1 complexes, inducing the inactivation of fibrinolysis and the prothrombotic state, which leads to the progression of PAD [142].

Acute ischemic stroke and acute intracerebral hemorrhagic stroke are among the main causes of mortality and morbidity worldwide, with a heavy healthcare and social global burden [143,144,145,146] Their complex pathophysiology involves several elements, such as oxidative stress, inflammatory response, mitochondrial dysfunction eventually leading to the activation of glial cells, the disruption of the blood–brain barrier, and microvascular disorders [147].

Above many serum biomarkers which are and can be detected during an acute stroke event, such as inflammatory markers (e.g., C-reactive protein, interleukin 6, and fibrinogen), circulating microRNAs (miRNAs) have recently been taken into account as potential biomarkers of the risk, severity, and characterization of acute stroke. MiRNAs are endogenous noncoding short single-stranded RNAs already known for their role in brain physiological development and function [148,149,150,151,152,153]. Giordano M. et al. reported a significant increase in circulating miRNAs during the acute phase of both intracerebral hemorrhagic stroke and ischemic stroke [154,155]. In particular, some studies have shown that miRNAs regulate several genes and pathways associated with ischemic stroke, including coagulation, platelets, and thrombus formation [156], and the modulation of their expression is implicated in neoangiogenesis and neurogenesis. In this regard, some of these miRNAs, specifically miRNA−195-5p and −451a, which are (HIF-1α)-induced miRNAs (HRMs), have been shown to target VEGF-A [157,158]. Thus, the high expression of these miRNAs seems to be related to low levels of VEGF-A and angiogenesis.

VEGF-A is a vascular remodeling mediator implicated in brain recovery and circulation after stroke, and its increased expression after the very first hours of an acute stroke event display a recovery phase.

In studies including acute ischemic-stroke patients, within 96 h of the acute event, a significant decline in circulating HRMs (−195-5p and −451a) was reported. This decline was correlated with incremented levels of VEGF-A, suggesting an attempt to recover from brain vascular damage [159,160]. In acute hemorrhagic stroke patients, the abovementioned miRNAs levels do not appear to decrease, with a specular non-incremental change in serum VEGF-A levels after 96 h [161]. Moreover, while a significant negative correlation has been observed between serum miRNA −195-5p and −451a expression and serum VEGF-A levels in acute ischemic stroke patients, no correlation has been found between these elements in hemorragic stroke patients.

These data could suggest a different recovery response over time between the two types of stroke involving the carotoid tree, and more studies implicating a longer follow-up might be needed. Surely, miRNAs might be potential clinical stroke biomarkers and a helpful tool to characterize stroke patients. For instance, Giordano M. et al. [162] found that diabetic patients with stroke showed a larger expression of miRNA-195-5p and miRNA-451a and a lower expression of VEGF-A levels in comparison to non-diabetic patients, probably reflecting a different risk-profile of diabetic patients who show a particularly complex interplay of several inflammatory and metabolic aspects, strongly affecting the cardio-vascular system [163,164].

## 10. The Role of Genetics in Peripheral Artery Disease

Many studies have been conducted to find a possible genetic substrate predisposing to PAD development, regardless of the role of behavioral risk factors and comorbidities.

### 10.1. Prothrombin G20210A Mutation

About 2–4% of the general population carries the prothrombin *G20210A* mutation, which is notably involved in a higher risk of deep vein thrombosis events. Therefore, the discovery that this mutation is highly prevalent in PAD patients suggested a possible role of this variant in the formation of atherosclerotic plaque and the progression of peripheral artery disease.

A past study (LIPAD) performed in 2000 excluded any association between genetic mutation and PAD [165]. However, some recent trials have revalued a possible interconnection between mutations in the genes coding for clotting factors and the risk of the development and progression of peripheral atherosclerotic disease.

A review carried out by Vazquez et al. showed a prevalence of the mutation of 4.5% in patients with LEAD compared to a lower prevalence (2.8%) in the healthy group, demonstrating non-significant differences between the two groups of the study. Furthermore, a statistically significant difference has been found in patients affected with LEAD and presenting with chronic limb ischemia: in these patients, a prevalence of the prothrombin *G20210A* of 7.6% was found, three times higher than that in the controls [166].

### 10.2. Factor V Leiden

Factor V is a soluble protein, circulating in plasma in an inactive form and activated after proteolysis mediated by the thrombin. Activated FV is a procoagulant factor that, binding factor Xa and calcium, constitutes a clotting complex mediating prothrombin activation into thrombin; aFV is degraded by aPC (activated protein C).

Factor V Leiden originates from a single-point mutation of the gene in position 506 (R506Q): the mutation removes the binding site for aPC, prolonging the half-life of the protein and resulting in the prolonged activation of the coagulation cascade. FV Leiden is well known to be associated with a higher risk of venous thromboembolic disease, but its role in atherosclerotic disease has yet to be established. In a recent study, FV Leiden has been associated significantly with the risk of LEAD: the variant has been found in patients with claudicatio intermittens, rest pain, tissue loss, and major amputation, with a higher effect in advanced disease, mostly in smokers [167].

### 10.3. Methylenetetrahydrofolate Reductase (MTHFR) C677T Mutation

MTHFR is an enzyme converting the 5,10-methylenetetrahydrofolate into the active form 5-methyltetrahydrofolate. The *C677T* mutation is associated with the substitution of a valine with alanine in position 677 of the protein, which provokes a loss of function of the enzyme and the reduction in homocysteine metabolism.

The Norwich case-control study was the first trial concerning the possible correlation between MTHFR *C677* mutation and PAD: the study concluded that the MTHFR *C677T* allele has a potential role in the risk of developing atherosclerosis, since the mutation in homozygosity (TT) was found to have a higher prevalence in PAD patients (with an odds ratio of 1.99) than in the control group, emphasizing, therefore, the strong association between PAD and the TT genotype [168].

## 11. Rational Therapy

Patients with PAD present dysregulated procoagulant, anticoagulant, and fibrinolytic pathways [169].

These elements, along with a pro-inflammatory milieu [170], provide the basis for atherosclerotic plaque and arterial thrombosis formation, leading to an increased risk and incidence of local and major ischemic events, resulting in augmented cardiovascular and cerebrovascular mortality [171]. Both historically and more recently screened therapeutic measures for secondary prevention are employed to counteract the abovementioned dysregulated pathways of platelet and coagulation factors activation.

Nowadays, antiplatelets represent the classical approach to limb-related and major cardiovascular events prevention in LEAD, and their use is currently recommended by the European Society of Cardiology (ESC) and the American Heart Association/American College of Cardiology (AHA/ACC) guidelines in: (i) patients with symptomatic Lower Extremity Artery Disease or who have undergone revascularization; (ii) all patients with carotid artery stenosis regardless of clinical symptoms and revascularization [172,173,174].

Therefore, key elements guiding physicians’ conduct towards the prescription of antithrombotic therapy are the affected district, the clinical presentation, and the contingent revascularization procedure. Other aspects should also guide the chosen therapeutic regimen: single or dual-antiplatelet therapy (DAPT) are both used to reduce the risk of atherothrombotic events. Single antiplatelet therapy (SAPT) usually involves the administration of aspirin or clopidogrel, while DAPT combines aspirin with a P2Y_12_ inhibitor, such as clopidogrel, ticagrelor, or prasugrel. If a concomitant indication is present, antiplatelet therapy can be considered in association with anticoagulant therapy. Currently, four major types of antiplatelet agents are used for the prevention and treatment of arterial thrombosis: acetylsalicylic acid, P2Y12 antagonists, αIIbβ3 (also known as gpIIb/IIIa) antagonists, and a Par1 inhibitor.

Acetylsalicilic acid is a nonsteroidal antinflammatory drug whose mechanism of action depends on the daily administered dose. In particular, ASA produces a relevant antiplatelet effect by irreversibly acetylating cyclooxygenase-1 (COX-1), which is required for the production of thromboxane A2, a promoter of aggregation, and these consequences are obtained by daily doses of 75 mg (and higher). Higher doses of aspirin also inhibit COX-2, which leads to analgesic and antipyretic effects [175].

P2Y_12_ inhibitors provide further suppression of platelet activity, as P2Y12 is the core receptor involved in the ADP-stimulated activation of the glycoprotein IIb/IIIa receptor. Antagonizing this receptor prevents its binding to ADP, resulting in attenuated platelet aggregation and the reaction of platelets to the stimuli of thrombus aggregation such as thrombin [176,177,178].

More recently developed P2Y12 inhibitors (i.e., prasugrel, ticagrelor, and cangrelor) are more potent and have a faster onset of action than clopidogrel [179].

With the blockade of the TXA2 pathway and the P2Y12 receptor, platelets can still be activated by thrombin via the PAR-1 receptor, with the aim of strengthening antiplatelet action. The TRA2°P-TIMI 50 trial studied the efficacy of vorapaxar, a protease-activated receptor-1 inhibitor (the primary receptor for thrombin on human platelets), in 26,449 patients with stable atherosclerotic vascular disease (myocardial infarction, stroke, or peripheral artery disease) and found that vorapaxar did not decrease the risk of major cardiovascular events in patients with peripheral artery disease but reduced the risk of acute limb ischemia and peripheral revascularization regardless of the mechanism of acute limb ischemia, native vessel thrombosis, or graft-related procedure thrombosis, although at the cost of an increased bleeding risk [180,181]. The favorable efficacy profile has determined FDA approval of vorapaxar in 2014 in patients with coronary artery disease or peripheral artery disease combined with single antiplatelet therapy or DAPT (excluding patients with a history of stroke or transient ischemic attack). However, despite its approval, vorapaxar is not frequently used, probably because of concerns regarding the increased bleeding risk [182].

Moreover, since PAR-1 on endothelial cells mediates mitogenic effects, vorapaxar might also be able to reduce vascular remodeling and, hence, the progression of atherosclerosis [180,182]. Although the prevention of atherothrombotic major events has conventionally been focused upon the inhibition of platelet aggregation [175], acute atherothrombotic events are caused by the disruption or erosion of atherosclerotic plaques and overlapping thrombosis, in which the concomitant activation of platelets and coagulation is needed [183].

The two pathways (coagulation cascade and platelet activation) intersect at several sites; for example, thrombin is a key element of the coagulation cascade as well as a robust inducer of platelet activation and aggregation via protease-activated receptors, such as PAR-1 and PAR-4, on the platelet surface [183]. Consequently, the factors implicated in thrombin activation, such as factor Xa, are implicated in this network. Moreover, recent studies suggest that factor Xa may also activate PAR-1 and that platelets may play an important part in amplifying thrombin generation, because the intrinsic enzyme complexes, which mediate the generation of factor Xa and thrombin, assemble on the activated platelet surface [184].

These assumptions explain how the antithrombotic strategy, which nowadays relies only on antiplatelet agents, may be inadequate to fiercely suppress atherothrombotic events, considering that, despite the standard of care therapies and its improvements, such as DAPT, the risk of major cardiovascular events remains high [185,186,187].

Hence, there is a need to investigate dual pathway inhibition strategies, where an antiplatelet agent is combined with an anticoagulant, whose role, despite pathophysiological premises and trials’ results, is still under debate.

Apart from actual guidelines’ recommendations, which suggest oral anticoagulant therapy if a concomitant indication is present (i.e., paroxysmal, persistent, or permanent Atrial Fibrillation with a CHA2DS2-VASc (Congestive heart failure, Hypertension, Age ≥ 75 (2 points), Diabetes mellitus, Stroke or TIA (2 points), Vascular disease, Age 65–74 years, Sex category) score ≥ 2; mechanical heart valve; recent or a history of recurrent deep venous thrombosis or pulmonary embolism) [188], several studies over recent years have been evaluating the putative role of oral anticoagulants in association with an antiplatelet agent (as an alternative to a mono or dual antiplatelet regimen) for the reduction of cardiovascular outcomes (both local and systemic events) [189].

Among the four major classes of anticoagulants used in clinical practice, heparins (parenteral), direct thrombin inhibitors, vitamin K antagonists (VKA), and direct Factor Xa inhibitors, the last two have been studied in order to be associated or used in LEAD patients.

Warfarin is a competitive inhibitor of vitamin K epoxide reductase, and its effect leads to a reduction in vitamin K, which is required for the post-translational modification of several proteins in the coagulation cascade requiring this modification to create a positively charged, active domain [190].

In 2007, Anand S. et al., in the WAVE trial [191], concluded that the combination of warfarin and antiplatelet therapy was not more effective than antiplatelet therapy alone in preventing major cardiovascular complications and was associated with an increase in life-threatening bleeding (life-threatening bleeding occurred in 43 patients receiving combination therapy (4.0%) as compared with 13 patients receiving antiplatelet therapy alone (1.2%) (relative risk, 3.41; 95% CI, 1.84 to 6.35; *p* < 0.001)). These findings surely pointed out the need to consider alternatives to vitamin K antagonists in patients with LEAD.

Direct oral anticoagulants (DOACs) bind to the active site of their target enzyme and block its activity. They produce a more predictable anticoagulant response than VKAs since their metabolism is unaffected by genetic polymorphisms or variations in dietary vitamin K intake, with fewer drug–drug interactions. Although other DOACs have been examined in patients with atherosclerotic disease, the majority of trials have been focused on rivaroxaban, an FXa inhibitor [192,193].

The first trial to study the efficacy and safety of DPI in patients with cardiovascular diseases was the “Anti-Xa Therapy to Lower cardiovascular events in Addition to aspirin with/without thienopyridine therapy in Subjects with Acute Coronary Syndrome” (ATLAS ACS 2-TIMI 51) trial, which demonstrated that the addition of rivaroxaban (2.5 or 5 mg twice daily) to SAPT (low-dose aspirin) or DAPT (aspirin plus a P2Y_12_ inhibitor) reduced atherothrombotic risk in patients with a recent acute coronary syndrome compared with antiplatelet therapy alone [194].

The first trial to study the use of rivaroxaban in patients with PAD was the COMPASS trial [195], which concluded that, in selected high-risk patients (polyvascular disease ≥ 2 vascular beds affected with atherosclerosis, impaired renal function (estimated glomerular filtration rate ≤ 60 mL/min), heart failure, diabetes mellitus, or a combination of these risk characteristics) suffering from chronic coronary syndromes and/or PAD, the combination of rivaroxaban 2.5 mg twice daily plus acetylsalicylic acid 100 mg reduced the risk of cardiovascular events as compared with aspirin monotherapy, even at an increased risk of major bleeding.

Capell et al., in the VOYAGE PAD study [186], concluded that rivaroxaban 2.5 mg twice daily with aspirin versus aspirin alone reduces first and subsequent adverse limb and cardiovascular events, with a greater total benefit when considering all events and with a significant reduction in the composite of acute local (limb) ischemia, major amputations for vascular causes, and major cardiovascular causes in patients in the rivaroxaban arm, even after 3 years of follow-up. The efficacy of rivaroxaban at a “vascular dose” in patients with chronic CAD and LEAD, in contrast to the high doses required for secondary cardiovascular prevention in patients with AF and venous thromboembolism, can be explained by the differences in the pathophysiology of these conditions and the reason for the need for a synergistic effect with antiplatelets: in venous thromboembolism and in the left atrium of patients with AF, thrombi mold under low-shear stress conditions mainly consists of fibrin [191]. In contrast, in atherothrombosis, thrombi form under high-shear conditions and are platelet-rich but thrombin-driven, thus indicating the need for an anticoagulant in addition to antiplatelet treatments, taking into account the increased risk of bleeding derived from their combination such that is important to use the lowest effective dose of anticoagulant to diminish the risk of bleeding [188,189].

The beneficial effects determined by factor Xa and thrombin inhibition in this setting are due not only to the attenuation of coagulation but also to the inhibition of thrombin-mediated platelet activation and the suppression of several processes leading to atherogenesis, vascular inflammation, and plaque instability. These beneficial effects of DOACs are thought to be caused by the inhibition of thrombin’s PAR-mediated signaling: since Factor Xa is thought to stimulate a pro-inflammatory milieu by directly activating PAR-2 receptors, the inhibition of factor Xa could attenuate thrombo-inflammation [10].

These three trials underline some important findings for the use of combined anticoagulant–antiplatelet in atherosclerotic disease as the potential new standard of care in LEAD.

## 12. Anti-Inflammatory and Immunomodulatory Drugs as a New Potential Therapeutic Strategy: A Hope for The Future?

Several studies have been performed to examine the possible efficacy of drugs blocking immune and inflammatory pathways as a future therapeutic strategy in patients with atherosclerotic disease.

### 12.1. ANTI-IL1 Agents

Regarding the essential role of IL-1 in the pathogenesis of plaque formation, anti-IL1 agents have been evaluated as a potential treatment in atheromasia in some phase III clinical trials.

In the CANTOS trial, the administration of Canakinumab, an antagonist of IL-1beta, reduced the recurrence of cardiovascular events in patients with a history of myocardial infarction and CRP values > 2 mg/L as compared to a placebo. Moreover, the decrease in CRP to values < 2 mg/L has been associated with more benefits in the primary outcome. The reported data underlined the strong correlation between inflammation and atherosclerosis [196]. In further studies, the efficacy of other anti-IL1 agents has been investigated, such as Anakinra, an antagonist of the IL-1 receptor and an inhibitor of IL-1α and IL-1β. A study by Morton et al. [197] showed a reduction in CRP after 14 days of treatment with anakinra but an increase in major adverse cardiac events (MACE) after 1 year of treatment in comparison with a placebo. These data have not been confirmed in a more recent trial [198], whose final results have shown a reduction in the incidence of new-onset heart failure and hospitalization and death for heart failure in the group of patients treated with anakinra.

No differences in vessel restenosis and the incidence of major adverse cardiovascular events have been found between placebo and Xilonix, a monoclonal antibody targeting IL-1α. [199] These controversial data raise questions about the effective role of anti-IL1 agents as modulators of the atherosclerotic processes.

### 12.2. Colchicine

The anti-inflammatory action of colchicine results from multiple processes, including the block of IL-1 release consequent to the inhibition of inflammasome, the inhibition of the microtubule polymerization, and the impairment of leukocyte activity. Furthermore, the COLCOT [200,201] trials highlighted the efficacy of colchicine treatment in reducing MACE in patients with previous acute cardiovascular events and chronic cardiac ischemia, suggesting a potential role in reducing atherosclerosis progression.

### 12.3. ANTI-IL6 Agents

Promising phase II clinical trials are underway for testing the efficacy of anti-IL-6 agents. Ziltivekimab and Tocilizumab, two monoclonal antibodies against IL6 and its receptor, have been associated with a reduction in CRP levels, showing the anti-inflammatory role of these drugs and suggesting a possible use of these molecules for the treatment of atherosclerotic disease [202,203,204], even if an increased risk of the occurrence of the MACE was observed in patients with rheumatoid arthritis treated with anti-IL6 [205]. However, more data need to be collected to confirm or disprove the clinical efficacy of anti-IL6 agents in preventing atherosclerosis progression.

### 12.4. ANTI-TNF Agents

Despite the prominent role of TNF in atherogenesis, no differences in atherogenesis and cardiovascular events have been detected in patients treated with anti-TNF compared to a placebo [206,207,208].

### 12.5. Immunomodulatory Drugs

Hydroxychloroquine is an immunomodulatory drug whose action explicates through the reduction in cyclic GMP–AMP synthase levels and the inhibition of Toll-like receptors, resulting in the decrease of pro-inflammatory cytokines such as INF [209]. Currently, hydroxychloroquineis is largely used as a disease-modifying antirheumatic drug in rheumatologic diseases, where the efficacy in reducing the rate of cardiovascular and thromboembolic events has been proven [210]. Moreover, hydroxychloroquine is the subject of ongoing studies evaluating a possible role in modifying the course of atherosclerotic disease [211]. IL-2 has been proposed as a potential treatment for inflammatory disease, considering its action on the proliferation of regulatory Treg cells. Preliminary data from ongoing studies [211] support this hypothesis.

Finally, several studies have investigated other possible targets such as the monocyte recruitment depending on CCR2, CCR5, and CX3CR1 chemokine receptors, [212,213] the modulation of the inflammatory power of macrophages through the regulation of several pathways (nuclear factor-κB, the STAT family, peroxisome proliferator-activated receptor-γ PPARγ, and the interferon regulatory factor family) [214], the inhibition of inflammasome [215], and the reprogramming of the adaptive immune cells activity [216,217,218,219]. They all represent possible pathways to target for future therapeutic option development.

Thus, despite the well-known link between inflammatory–immune pathways and atherosclerosis, the research has failed to submit compelling results to support the efficacy of anti-inflammatory and immunomodulatory drugs for treating atherosclerosis. At the current state, more studies need to be carried out.

## 13. Future Perspectives

Antiplatelet agents represent the standard of care in clinical practice. Nevertheless, recent studies showed that DOACs may be a good therapeutic choice for thromboprophylaxis in PAD, but with an increased bleeding risk. Thus, research of antiplatelets and anticoagulation alternatives with better safety profiles has been focusing on other components of the hemostasis system [180,220]. The improved understanding of the mechanisms of hemostasis and thrombosis has revealed new possible targets with the potential for less of a bleeding risk, some of which are currently under evaluation in phase III trials, although mostly in the context of CAD.

The potential roles of vorapaxar and the PAR-1 inhibitor have already been discussed. PAR-1 is a G protein-coupled receptor found on the platelet surface, and its cleavage is a thrombin target to activate platelets; although having received approval from the Food and Drug Administration, vorapaxar is a slowly reversible inhibitor and could interfere with the physiological thrombin-mediated activation of platelets during hemostasis, leading to an increased bleeding risk [180].

PZ-128 is part of the “PAR-1 inhibitors family”, is a pepducin, and is hence a lyophilic molecule which targets cytoplasmatic domains of the receptor. Its safety, efficacy, and pharmacokinetic characteristics were evaluated in a phase I study in patients with vascular disease or who had two or more CAD risk factors: it was found to be a promising antiplatelet agent, providing the rapid, specific, dose-dependent, and reversible inhibition of PAR-1 with no effects on bleeding, coagulation, or clinical chemistry [221].

While PAR-1 inhibition acts on thrombin-induced platelet adhesion during plug formation (acute event), PAR-4 inhibition acts upon platelet cohesion during the atherothrombosis chronic process [222].

Among various molecules, BMS-986120 was found to be a potent and reversible PAR4-specific small molecule antagonist, and, compared to clopidogrel, it decreased the thrombus load to a similar level, but, unlike clopidogrel, had little effect on the bleeding time and did not cause spontaneous bleeding when administered to healthy volunteers [180,223].

Glycoprotein VI (GPVI) represents another interesting new target for antiplatelet therapy; it is the platelets’ main receptor for collagen and also binds fibrin [224,225]. Ruptured atherosclerotic plaques are rich in collagen, thus providing a solid area for GPVI-mediated platelet adhesion, although patients lacking GPVI present a minor predisposition to bleeding. These assumptions suggest that acting on GPVI might be useful for preventing acute events determined by the rupture of atherosclerotic plaques, so some agents, operating on GPVI itself (ACT017) or on GPVI ligand collagen (ravacapt), have been developed [226]. Although positive safety and tolerability profiles have been demonstrated, no results have been reported in phase II trials [227,228].

Furthermore, platelets’ attraction to the injured vessel is ensured by the interaction of the platelet receptor glycoprotein Ibα (GPIbα) with VWF, which is exposed on the extracellular matrix, and this interaction was found to be important under high-sheer stress conditions, taking place in arterial thrombosis [229,230].

Some investigated VWF inhibitors include ARC1779, a DNA-based aptamer, two monoclonal antibodies (AJW200 and 82D6A3), and two bivalent nanobodies (caplacizumab (ALX-0081) and ALX-0681), and among these, caplacizumab was found to be well tolerated and without an increased bleeding predisposition in two phase I clinical trials (NCT0289733 and NCT03172208). Similarly, various GPIbα inhibitors have been tested, although the loss of GPIbα is associated with increased platelet clearance, which is an unwanted effect that must be avoided [231].

Stable platelet adhesion under high-shear stress conditions is guaranteed by phosphoinositide 3-kinase-β (PI3Kβ), which is pivotal in signaling pathways of various platelet receptors [232].

AZD6482, a PI3Kβ inhibitor, was well tolerated in healthy volunteers in a phase I trial (NCT00688714), and the ASA–AZD6482 association was found to have a stronger antiplatelet activity and less bleeding compared to the ASA–Clopidogrel association in DAPT [233,234].

Studies have also focused on the coagulation pathway, considering that currently used anticoagulants directly or indirectly target components of the common pathway (Factor Xa) which are essential for physiological hemostasis and whose inhibition carries a non-indifferent bleeding risk.

The studies to date in this field have shown interesting results in examining other components of the contact system with a potentially more favorable safety profile [183].

Factors of the intrinsic pathway (mainly FXII, FXI, and FIX) are, for example, appealing targets for the development of novel anticoagulants because their inhibition could cause lower bleeding compared with anticoagulants that target a common pathway [235,236].

Some of the tested molecules, i.e., TTP889, an FIXa inhibitor, and SB249417, a monoclonal antibody targeting FIX, although promising in in vitro results [237,238], have not been further explored due to a lack of results.

The most surprising results come from targeting FXI and FXII. The used molecules are antisense oligonucleotides, monoclonal antibodies, aptamers, and small molecules [239,240].

For instance, a preclinical study evaluated the antithrombotic activity of the Ixodesricinus contact phase inhibitor (Ir-CPI), a protein that inhibits both FXIIa and FXIa, in initiated thrombosis animal models. Interestingly, during cardiopulmonary bypass, Ir-CPI was found to be as efficient as unfractionated heparin in preventing clot formation within the extracorporeal circuit and maintained physiological parameters during and after surgery without increasing bleeding risk [241,242,243].

In the AXIOMATIC-SSP trial, a phase II trial for secondary prevention in patients with stroke or high-risk TIA to be completed this year (2022), BMS 986177, an oral FXIa inhibitor, has been administered in association with ASA and clopidogrel (or a placebo) for 21 days followed by aspirin alone, with the composite primary outcome of a new ischemic stroke during the treatment period and a new covert brain infarction detected by brain imaging at 90 days [244].

Regarding the atherosclerotic disease, the PACIFIC-AMI (NCT04304534) trial will compare the efficacy and safety of BAY2433334, another oral FXIa inhibitor in association with DAPT, with a placebo for the prevention of major adverse cardiovascular events in 1600 patients with acute myocardial infarction (results are expected for 2022) [245].

Other innovative approaches consist in blocking FXII activation by blocking its activators; in particular, nucleic acid scavengers and polyphosphate inhibitors have been shown to reduce thrombosis in mouse models, although, to date, none of these agents have been tested in trials studying their effects in thrombi formation [246,247].

## 14. Conclusions

The cross-talking between inflammatory molecular pathways, the coagulation system, and the artery wall represents an important substrate for the development and thrombogenic progression of atherosclerotic lesions in PAD. Coagulation acts on several levels, intersecting and acting in synergy with the inflammatory pathways. Nevertheless, the apport of the coagulation profile needs to be deeply investigated to elucidate the phenomenon of atherothrombotic occlusion associated with a non-significant intimal inflammatory pathology in PAD. The activation of the coagulation and inflammatory pathways appears to differ according to the location of the plaques (coronary or peripheral) and the characteristics of the plaque itself. It is likely that the specific interactions between the arterial wall, inflammatory molecules, and coagulation in each type of plaque can be an important determinant in the histopathological composition of atherosclerosis and in its future stability. New studies to assess the influence of coagulation and the immune system on the atherogenesis of peripheral plaques will probably allow for the identification of more plaque subtypes with different characteristics that are responsible for different clinical scenarios. Understanding the role of coagulation and the immune system in atherosclerosis genesis and progression could help in choosing the right patients for future possible anti-inflammatory and dual pathway inhibition strategies.

## Data Availability

Not applicable.

## Abbreviations

ABI: Ankle Brachial Index; aPC: Activated Protein C; CAD: Coronary Artery Disease; COX: cyclooxygenase; DAPT: dual-antiplatelet therapy; DOACs: Direct oral anticoagulants; LEAD: Lower Artery Disease; MACE: major adverse cardiac events; PAD: Peripheral Artery Disease; PAR: protease-activated receptors; SAPT: Single antiplatelet therapy; TF: Tissue Factor; TFPI: Tissue Factor Pathway Inhibitor; tPA: Tissue Plasminogen Activator; VKA: Vitamin K Antagonists; VWF: von Willebrand’s factor.
